# Supplementary material for: Selective O2/N2 Separation Using Grazyne Membranes: A Computational Approach Combining Density Functional Theory and Molecular Dynamics
Source: Nanomaterials (Basel). 2024 Dec 22;14(24):2053. doi: 10.3390/nano14242053 (PMC11677545; doi:10.3390/nano14242053)
Supplement: Supplementary file 1 [file nanomaterials-14-02053-s001.zip › nanomaterials-3365889-supplementary.pdf]

Selective O_2/N_2 Separation Using Grazyne Membranes: A Computational Approach Combining Density Functional Theory and Molecular Dynamics

Adrià Calzada, Francesc Viñes and Pablo Gamallo *

Departament de Ciència de Materials i Química Física, Institut de Química Teòrica i Computacional (IQTCUB), Universitat de Barcelona, C/Martí i Franquès 1-11, 08028 Barcelona, Spain
* Correspondence: gamallo@ub.edu

Contents

Section S1. Vibrational modes of the adsorbed O_2/N_2 molecules S2
Table S1. Frequencies of perpendicular O_2 on [1],[2]{2}-grazyne S2
Table S2. Frequencies of parallel O_2 on [1],[2]{2}-grazyne S2
Table S3. Frequencies of perpendicular N_2 on [1],[2]{2}-grazyne S3
Table S4. Frequencies of parallel N_2 on [1],[2]{2}-grazyne S3
Table S5. Frequencies of perpendicular O_2 on [1],[2]{(0,0),2}-grazyne S3
Table S6. Frequencies of parallel O_2 on [1],[2]{(0,0),2}-grazyne S4
Table S7. Frequencies of perpendicular N_2 on [1],[2]{(0,0),2}-grazyne S4
Table S8. Frequencies of parallel N_2 on [1],[2]{(0,0),2}-grazyne S5
Section S2. Molecular Dynamics Simulations Videos for Grazyne Membranes S6
Trajectory S1. MD trajectory of the O_2/N_2 mixture on the [1],[2]{2}-grazyne S6
Trajectory S2. MD trajectory of the O_2/N_2 mixture on the [1],[2]{(0,0),2}-grazyne.S6

Section S1. Vibrational modes of the adsorbed O_2/N_2 molecules

This section provides detailed information on the vibrational modes of the adsorbed N_2 and O_2 molecules on the grazyne surfaces. It includes both qualitative descriptions and quantitative data, such as the vibrational frequencies and mode decompositions, which were used to calculate the adsorption and diffusion rates. These results provide deeper insights into the molecular behavior on the grazyne surface and complement the analyses presented in the main manuscript.

O_2 on [1],[2]{2}-grazyne

Table S1. Frequencies of O_2 (TS and adsorbed states) on the [1],[2]{2}-grazyne for the perpendicular conformation. Frequencies are shown in cm^{-1}, with associated motions labeled as stretching, rotation, vibration, or translation.

**Perpendicular**			
**TS (cm^{-1})**		**ADS (cm^{-1})**	
1355.686053	Stretching	1342.643691	Stretching
119.562135	Rotation	101.165885	Vibration
60.963528	Translation	80.130936	Vibration
28.931101	Translation	54.841435	Vibration
8.077322	Translation	42.075164	Vibration
-17.440670	Rotation	31.474793	Vibration

Table S2. Frequencies of O_2 (TS and adsorbed states) on the [1],[2]{2}-grazyne for the parallel conformation. Frequencies are shown in cm^{-1}, with associated motions labeled as stretching, rotation, vibration, or translation.

**Parallel**			
**TS (cm^{-1})**		**ADS (cm^{-1})**	
1332.297309	Stretching	1346.113854	Stretching
165.083085	Rotation	124.933205	Vibration
138.830258	Translation	103.519037	Vibration
62.928651	Translation	103.242171	Vibration
29.977911	Rotation	88.453114	Vibration
-49.723631	Translation	50.868880	Vibration

N_2 on [1],[2]{2}-grazyne

Table S3. Frequencies of N_2 (TS and adsorbed states) on the [1],[2]{2}-grazyne for the perpendicular conformation. Frequencies are shown in cm^{-1}, with associated motions labeled as stretching, rotation, vibration, or translation.

Perpendicular			
TS (cm^{-1})		ADS (cm^{-1})	
2434.130530	Stretching	2515.037750	Stretching
267.446458	Translation	159.854231	Vibration
223.544348	Rotation	158.776908	Vibration
199.423766	Translation	36.843696	Vibration
167.328445	Rotation	30.293400	Vibration
-141.468411	Translation	16.814911	Vibration

Table S4. Frequencies of N_2 (TS and adsorbed states) on the [1],[2]{2}-grazyne for the parallel conformation. Frequencies are shown in cm^{-1}, with associated motions labeled as stretching, rotation, vibration, or translation.

Parallel			
TS (cm^{-1})		ADS (cm^{-1})	
2432.987834	Stretching	2427.106732	Stretching
241.778273	Translation	180.484847	Vibration
201.722272	Translation	157.790752	Vibration
195.646513	Rotation	119.030756	Vibration
160.250393	Rotation	98.781117	Vibration
-78.692628	Translation	94.307362	Vibration

O_2 on [1],[2]{(0,0),2}-grazyne

Table S5. Frequencies of O_2 (TS and adsorbed states) on the [1],[2]{(0,0),2}-grazyne for the perpendicular conformation. Frequencies are shown in cm^{-1}, with associated motions labeled as stretching, rotation, vibration, or translation.

**Perpendicular**			
**TS (cm^{-1})**		**ADS (cm^{-1})**	
1375.253802	Stretching	1366.920199	Stretching
107.340162	Rotation	124.915194	Vibration
70.354485	Translation	61.984327	Vibration
50.380655	Translation	58.359963	Vibration
28.351859	Translation	50.951747	Vibration
-47.976123	Rotation	45.194612	Vibration

Table S6. Frequencies of O_2 (TS and adsorbed states) on the [1],[2]{(0,0),2}-grazyne for the parallel conformation. Frequencies are shown in cm^{-1}, with associated motions labeled as stretching, rotation, vibration, or translation.

**Parallel**			
**TS (cm^{-1})**		**ADS (cm^{-1})**	
1340.652265	Stretching	1376.338298	Stretching
183.146231	Rotation	84.443900	Vibration
155.206880	Translation	71.265580	Vibration
122.492237	Translation	64.621815	Vibration
81.835005	Rotation	58.107359	Vibration
-32.662079	Translation	20.839343	Vibration

N_2 on [1],[2]{(0,0),2}-grazyne

Table S7. Frequencies of N_2 (TS and adsorbed states) on the [1],[2]{(0,0),2}-grazyne for the perpendicular conformation. Frequencies are shown in cm^{-1}, with associated motions labeled as stretching, rotation, vibration, or translation.

Perpendicular			
**TS (cm^{-1})**		**ADS (cm^{-1})**	
2434.162276	Stretching	2436.930693	Stretching
243.979110	Translation	79.286320	Vibration
208.708252	Rotation	72.251457	Vibration
199.684129	Translation	67.095006	Vibration
156.996081	Rotation	28.173629	Vibration
-144.860907	Translation	18.352855	Vibration

Table S8. Frequencies of N_2 (TS and adsorbed states) on the [1],[2]{(0,0),2}-grazyne for the parallel conformation. Frequencies are shown in cm^{-1}, with associated motions labeled as stretching, rotation, vibration, or translation.

Parallel			
**TS (cm^{-1})**		**ADS (cm^{-1})**	
2429.921576	Stretching	2427.936282	Stretching
157.736372	Translation	213.641619	Vibration
122.023617	Rotation	170.621282	Vibration
115.936205	Translation	167.626870	Vibration
47.286314	Rotation	133.031500	Vibration
-108.817991	Translation	-78.347055	Vibration

Section S2. Molecular Dynamics Simulations Videos for Grazyne Membranes

Two videos of the molecular dynamics (MD) simulations for both grazyne structures are provided in the Supporting Information. These videos visualize the diffusion process of N_2 and O_2 molecules through the membranes, allowing the reader to observe the distinct behaviors of the molecules in both systems.

Trajectory S1: Molecular dynamics trajectory of the O_2/N_2 mixture on the [1],[2]{2}-grazyne with an initial gas pressure of 30.6 atm.

Trajectory S2: Molecular dynamics trajectory of the O_2/N_2 mixture on the [1],[2]{(0,0),2}-grazyne with an initial gas pressure of 31.0 atm.
